# Targeting Bacterial Cardiolipin Enriched Microdomains: An Antimicrobial Strategy Used by Amphiphilic Aminoglycoside Antibiotics

**DOI:** 10.1038/s41598-017-10543-3

**Published:** 2017-09-06

**Authors:** Micheline El Khoury, Jitendriya Swain, Guillaume Sautrey, Louis Zimmermann, Patrick Van Der Smissen, Jean-Luc Décout, Marie-Paule Mingeot-Leclercq

**Affiliations:** 1 Université catholique de Louvain, Louvain Drug Research Institute, Pharmacologie Cellulaire et Moléculaire, avenue E. Mounier 73, UCL B1.73.05, 1200 Brussels, Belgium; 2 Université Grenoble Alpes, Joseph Fourier/CNRS, Institut de Pharmacochimie Moléculaire, rue de la Chimie, F-38041 Grenoble, France; 3 Université Catholique de Louvain, de Duve Institute, avenue Hippocrate 75, UCL B1.75.05, 1200 Brussels, Belgium; 4 Present Address: Université de Lorraine, UMR CNRS UL 7565, 1 Blvd. Des Aiguillettes, BP 70239, 54506 Vandoeuvre-lès-Nancy Cedex, Nancy, France

## Abstract

Some bacterial proteins involved in cell division and oxidative phosphorylation are tightly bound to cardiolipin. Cardiolipin is a non-bilayer anionic phospholipid found in bacterial inner membrane. It forms lipid microdomains located at the cell poles and division plane. Mechanisms by which microdomains are affected by membrane-acting antibiotics and the impact of these alterations on membrane properties and protein functions remain unclear. In this study, we demonstrated cardiolipin relocation and clustering as a result of exposure to a cardiolipin-acting amphiphilic aminoglycoside antibiotic, the 3′,6-dinonyl neamine. Changes in the biophysical properties of the bacterial membrane of *P*. *aeruginosa*, including decreased fluidity and increased permeability, were observed. Cardiolipin-interacting proteins and functions regulated by cardiolipin were impacted by the amphiphilic aminoglycoside as we demonstrated an inhibition of respiratory chain and changes in bacterial shape. The latter effect was characterized by the loss of bacterial rod shape through a decrease in length and increase in curvature. It resulted from the effect on MreB, a cardiolipin dependent cytoskeleton protein as well as a direct effect of 3′,6-dinonyl neamine on cardiolipin. These results shed light on how targeting cardiolipin microdomains may be of great interest for developing new antibacterial therapies.

## Introduction

Gram-negative bacterial cell wall stability and plasmic membrane structural and functional integrity are crucial for the maintenance of bacterial shape and cellular functions. *Escherichia coli* (*E*. *coli*) L-spheroplasts, cells lacking cell wall, can divide and regenerate their rod shape once cell wall components are resynthesized^1^. In addition to its critical role for osmotic protection of bacteria^2^, cell wall is essential for conserving bacterial shape^3, 4^. Regarding lipid bilayers, they are the support of multiple cellular functions involving protein folding, structure, and function^5, 6^ preserving bacterial viability.

Functions of proteins are highly regulated^7^ including by lipids. Some of them play a critical role as demonstrated for cardiolipin (CL), a non-bilayer anionic phospholipid characterized by four acyl chains and small headgroup^8^. Cardiolipin is mostly located in mitochondria of eukaryotic cells and within the inner membrane of bacterial cells. Cardiolipin organizes into microdomains and cardiolipin interacting proteins are involved in fundamental and vital processes namely respiratory chain^9, 10^. Cardiolipin is possibly involved in a proton uptake pathway and in ensuring the structural integrity of related enzymatic complexes including cytochrome c oxidase and succinate-unbiquinone oxydoreductase^9–12^. Based on these data, cardiolipin and cardiolipin microdomains targeting compounds could be interesting to fight the increasing bacterial resistance to conventional antibiotics.

In previous studies, we showed that one of the most promising amphiphilic aminoglycoside derivatives synthesized by Decout’s team^13–15^, the 3′,6-dinonyl neamine (diNn) had bactericidal effect on a wide range of wild type Gram-negative and Gram-positive bacteria as well as resistant strains. On wild type *P*. *aeruginosa*, the minimal inhibitory concentration (MIC) was low (4 µg/mL). diNn also inhibits biofilm formation^16^. By using membrane model systems, we demonstrated the interaction of the amphiphilic neamine derivative with outer membrane’s lipopolysaccharides^16^ and inner membrane’s anionic phospholipids mostly cardiolipin leading to membrane permeabilization and depolarization^17^.

In this work, we report how a promising amphipihilic aminoglycoside derivative, the 3′,6-dinonyl neamine, by targeting cardiolipin-bacterial microdomains mainly located at the cell poles, leads to microdomains disassembly into cardiolipin clusters and relocation of cardiolipin domains as confirmed by using membrane model systems mimicking inner membrane of *P*. *aeruginosa*. Importantly, targeting cardiolipin-lipid domains results in bacterial morphological changes. These are characterized by a severe length decrease probably as a result from the decrease of the inner membrane fluidity/hydration induced by 3′,6-dinonyl neamine which could impair the dynamics of cell-shape determining proteins like MreB. Targeting cardiolipin could also explain the inhibition of the redox chain accompanied with an ﻿intracellular ATP concentration and pH decrease, and inhibition of cell growth induced by the amphiphilic neamine derivative.

From these results, we propose a new general mechanism for explaining the antimicrobial effect of amphiphilic aminoglycoside antibiotics able to interact with bacterial cardiolipin microdomains. These data could help to design on a rational basis promising lead compounds to fight antimicrobial resistance.

## Specific Interaction with cardiolipin disassembles bacterial CL microdomains into patches

**Figure 1:**
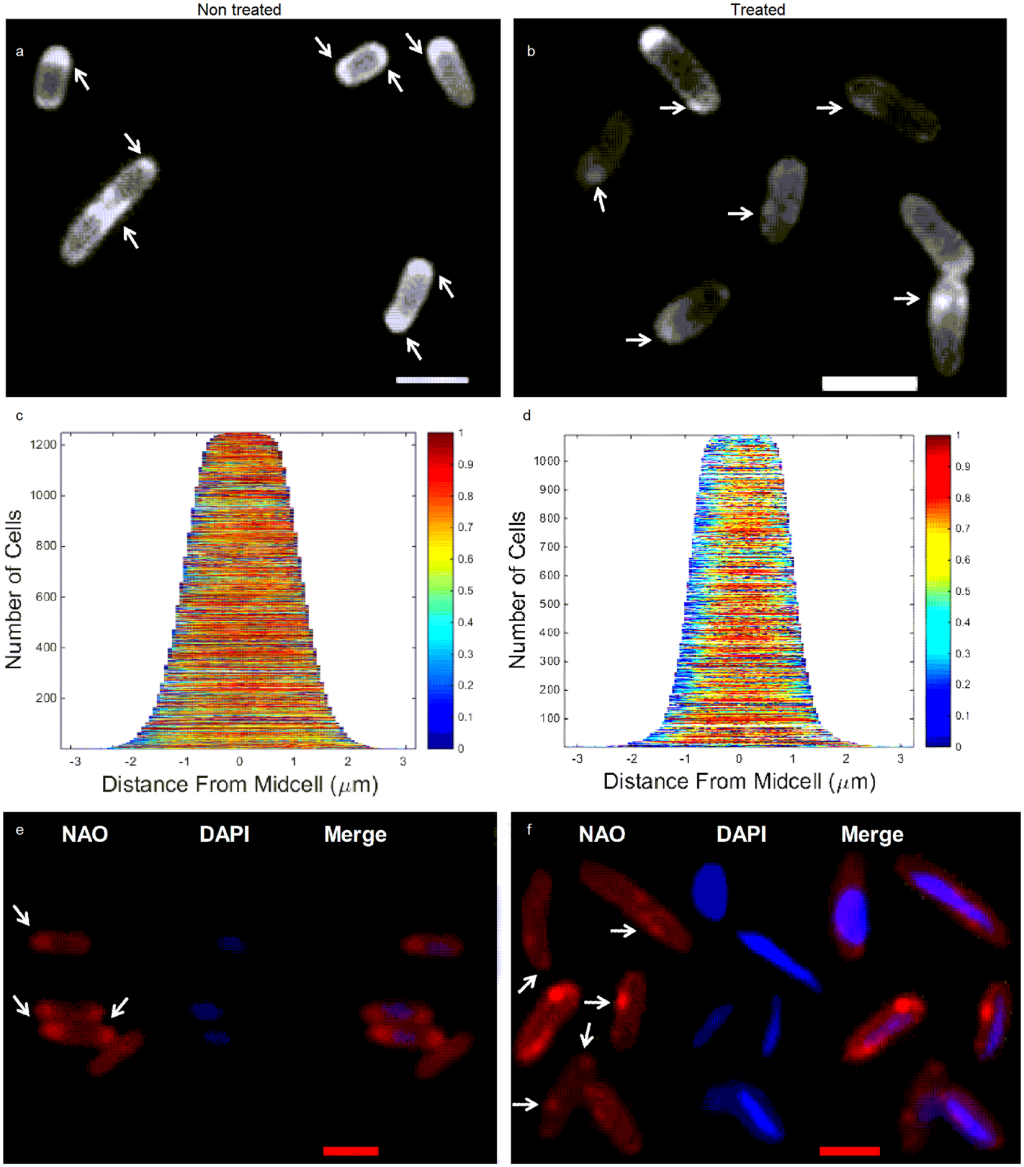
diNn targets *P*. *aeruginosa* microdomains of cardiolipin leading to their redistribution. Bacteria were incubated with 5 µM of diNn for 10 min at 37 °C. (**a**) Fluorescence images of TF-CL in control cells or (**b**) treated cells; arrows indicate CL microdomains as revealed by enriched areas with TF-CL. (**c**) Demographs representing axial signal profiles of TF-CL in control *P*. *aeruginosa* cells and (**d**) treated cells. (**e**) Fluorescence images of *P*. *aeruginosa* CL microdomains as revealed by NAO in control and (**f**) treated cells; arrows indicate CL microdomains. Nucleoids were visualized using 100 µM DAPI. Scale bars in **a**, **b**, **e**, **f** correspond to 2 µm. (N = 3)

In order to reveal the specific interaction between cardiolipin and diNn in bacteria, *P*. *aeruginosa* cells were enriched with TopFluor- cardiolipin (TF-CL) and incubated in the presence or absence of diNn. Figure 1 shows TF-CL lateral distribution pattern throughout non treated (Fig. 1a) or treated cells (Fig. 1b). It can be noticed that TF-CL was inserted throughout the bacterial membrane and patches were observed mainly at cell poles and septa. After incubation with diNn, the cardiolipin domains were disassembled and CL clusters were observed in regions neighboring cell poles. Demographs showing the axial distribution of fluorescence within the cells (Fig. 1c,d) confirm the redistribution of TF-CL after the incubation with diNn. When compared to control cells, an overall change in fluorescence distribution in cells was detected but the most important effect was seen at the cell poles suggesting a preferential interaction of diNn with cardiolipin-enriched areas. In this regard, microscale thermophoresis binding analysis demonstrated cooperative binding^18^ of diNn to TF-CL with a Hill coefficient of 3.4 (Data not shown).

In order to confirm the redistribution of cardiolipin induced by diNn, we used 10-*N*-nonyl acridine orange (NAO). This dye has been used to visualize CL enriched microdomains localized mainly at the polar and septal regions^19^ in *E*. *coli* ^20, 21^, *P*. *putida* ^22^, and *B*. *subtilis* ^23^. NAO has higher affinity to cardiolipin than other anionic phospholipids and its association with cardiolipin induces a red shift in its fluorescence emission^24, 25^. Cardiolipin microdomains were observed in *P*. *aeruginosa* control cells at the cell poles and septa region (Fig. 1e) whereas cardiolipin clusters were delocalized and are absent from the zones of high curvature, as a result of the presence of diNn (Fig. 1f). Together with results obtained with TF-CL, these observations suggest that diNn induces cardiolipin relocation within bacterial membrane.

## Targeting cardiolipin microdomains induces lipid rearrangements, enhances lateral phase separation and disrupts membrane biophysical properties

**Figure 2:**
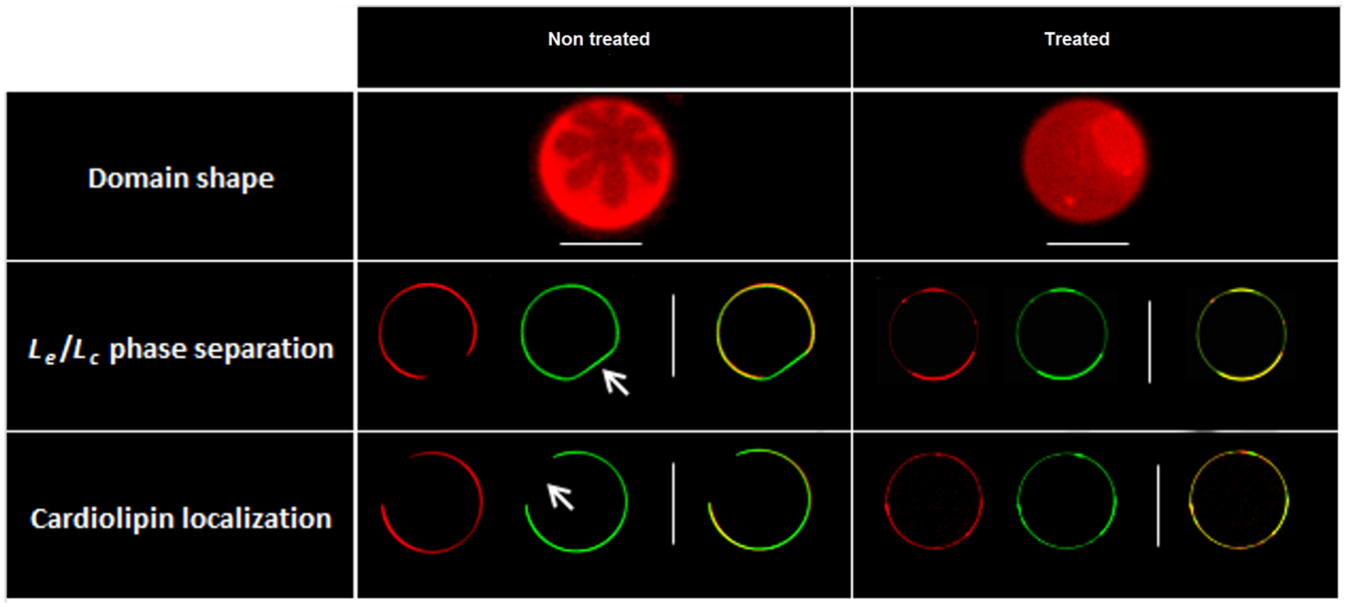
diNn induces modifications of lipid domains shape, enhancement of lipid phase separation and PE and CL segregation. Imaging of GUVs composed of PE/PG/CL in the absence (**a**) and in the presence (**b**) of diNn. Top: visualization through epifluorescence microscopy of lipid domain shape using the red TR-PE. Middle: visualization through confocal microscopy of L_*e*_/L_*c*_ phase separation using TR-PE and NBD-PE (green), respectively. Bottom: visualization through confocal microscopy of PE and CL localization using TR-PE and TF-CL (green), respectively. Arrows indicate a region in control GUVs without CL with a decreased curvature. White scale bars correspond to 10 µm. (N = 3).

**Figure 3:**
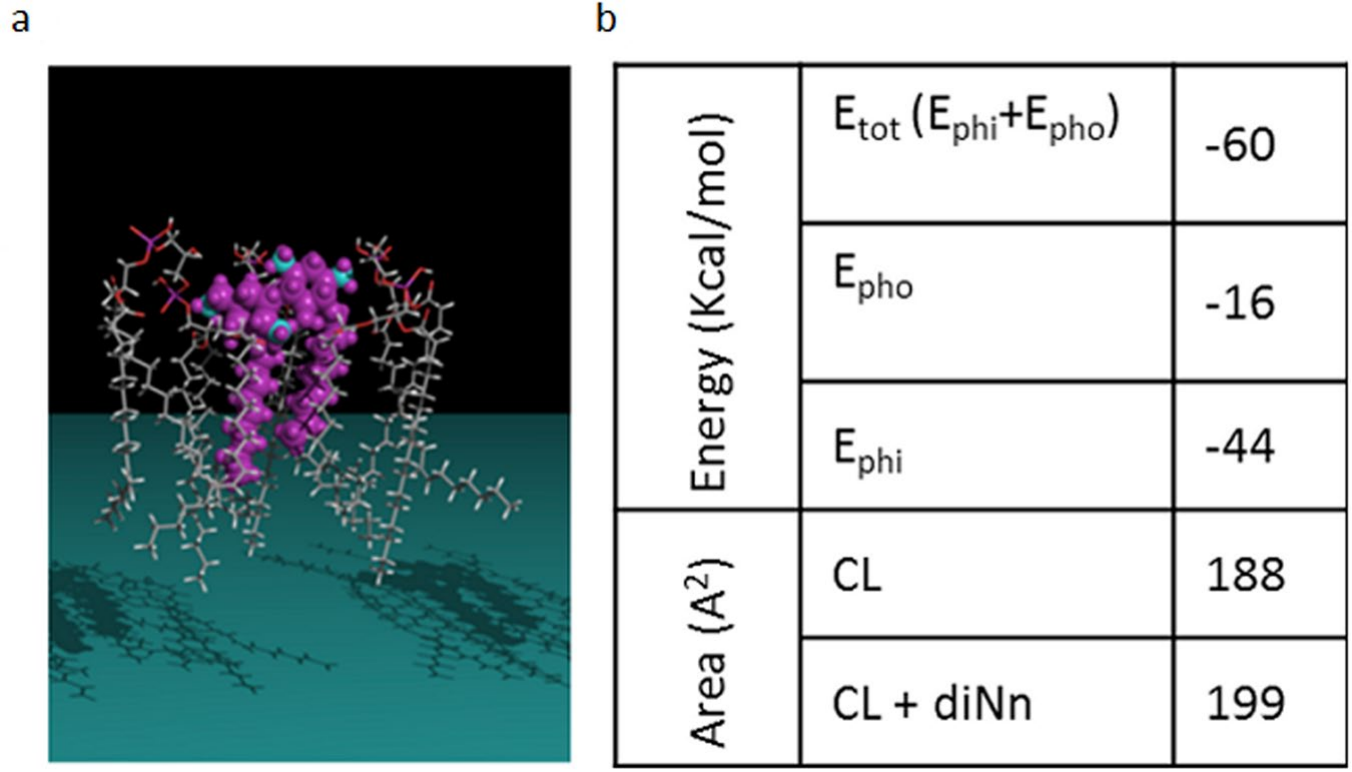
diNn interacts with CL through polar and electrostatic interactions. (**a**) Assembly of diNn with CL (left). The Neamine derivative in mauve is represented in real volume, nitrogen atoms are in blue. Lipids are in a skeleton representation. (**b**) Calculated energies of the interaction between the diNn molecule and cardiolipin tested experimentally. E_phi_ corresponds to polar and electrostatic interactions; E_pho_ corresponds to Van der Waals and hydrophobic interactions. Calculated mean interfacial area (Å^2^) of the lipid monolayer in the presence and in the absence of diNn.

Microdomains are in part the result of lipid distribution and lipid phase separation^26^. To investigate deeper the effects of diNn on lipid lateral phase separation, phosphatidyl ethanolamine / phosphatidylglycerol / cardiolipin (PE/PG/CL) Giant Unilamellar Vesicles (GUVs), mimicking inner membrane composition of *P*. *aeruginosa* ^27^ were labelled with texas red phosphatitylethanolamine (TR-PE) and *N*-(7-Nitrobenz-2-Oxa-1,3-Diazol-4-yl)-1,2-Dihexadecanoyl-sn-Glycero-3- Phosphoethanolamine (NBD-PE). The presence of TR-PE indicates a very fluid liquid expanded domain (*l*_e_), marking with solely NBD-PE indicates a more condensed liquid domain (*l*_c_) and the absence of any probe is due to a very compact gel phase (solid ordered, *s*_o_, domain)^28–31^. In the control condition (Fig. 2a), flower-shaped domains in addition to a *l*_e_/*l*_c_ phase separation as shown with TR-PE and NBD-PE labelling were observed (top). A domain labelled solely by NBD-PE with decreased curvature and without TF-CL was also observed (middle). Due to the negative spontaneous curvature of PE compared to PG^32^, it is most likely that it is a PE enriched phase. Moreover, CL was evenly distributed in the more fluid *l*_e_ phase marked by the TR-PE probe (bottom). In the presence of diNn (Fig. 2b), the initially flower-shaped domains disappeared and increased to surround smaller, round and more fluid domains (top). A shift in lipid phase separation and an increase in the fraction of more ordered domains were also detected (middle). Interestingly, diNn induces an increase in membrane curvature. Domains with decreased curvature labelled solely with TR-PE, and without cardiolipin in the control condition (showed with white arrows) were replaced by domains with increased curvature and containing cardiolipin. Cardiolipin localization studies (bottom) showed that after incubation with diNn, cardiolipin remained in the *l*_e_ phase. Clusters, sections with increased membrane curvature, enriched with cardiolipin and phosphatidylethanolamine were formed^17^. When changes of lipid packing occur, defects at the membrane interface are triggered, and favored mostly in coexisting gel-fluid domains governed by an equilibrium state between hydrophobic interactions within the hydrocarbon chains and head group repulsions at the interface, enriched in this case with negative charges of cardiolipin^33^. A concerted mechanism of induction of phosphatidylethanolamine-enriched low curvature phase and cardiolipin-clustering may be present. In biological membranes, lipids are concentrated at the domains boundaries in order to reduce the tension at the interface and stabilize membrane structure. When exogeneous compounds like the antibacterial derivative diNn induce the formation of anionic lipid clusters with modified curvature, membrane loses its ability to rearrange in order to accommodate these changes, leading to alteration of the stability and composition of lipid domains accompanied with membrane permeabilization and defects of bacterial functions^19^. Data obtained from GUV studies confirm the hypothesis suggesting that diNn induces cardiolipin reorganization and microdomains alteration through a shift in lipid phase separation and cardiolipin clustering. Molecular modelling calculations were conducted in order to study the interaction between diNn and cardiolipin (Fig. 3). Interaction results from both the polar electrostatic energy - the two positive charges of 3′,6-diNonyl Neamine are at 3–4 Å from the negative charge of cardiolipin (a distance sufficient for an electrostatic bond) and hydrophobic interaction (approximately 30 kcal/mol for Neamine and approximately 16 kcal/mol for the 3′,6-diNonyl Neamine). Concerning the mean area occupied by the lipids in the monolayer, we observed a significant increase for cardiolipin interacting with the diNn. All together, these simulation data suggest that interaction of diNn with cardiolipin is driven by electrostatic forces accompanied with an insertion into the lipid monolayer (Fig. 3). Also, an increase in the zeta potential of PE/PG/CL liposomes was observed in the presence of diNn (SI, Fig. S1) reflecting changes in the slipping plane which determines the Zeta potential^34^. Essentially, the interaction of diNn with cardiolipin and possibly with other negatively-charged phospholipids induces changes in the distribution of counter ions with their hydration shell at the membrane aqueous interphase directly linked to the Zeta potential.

**Figure 4:**
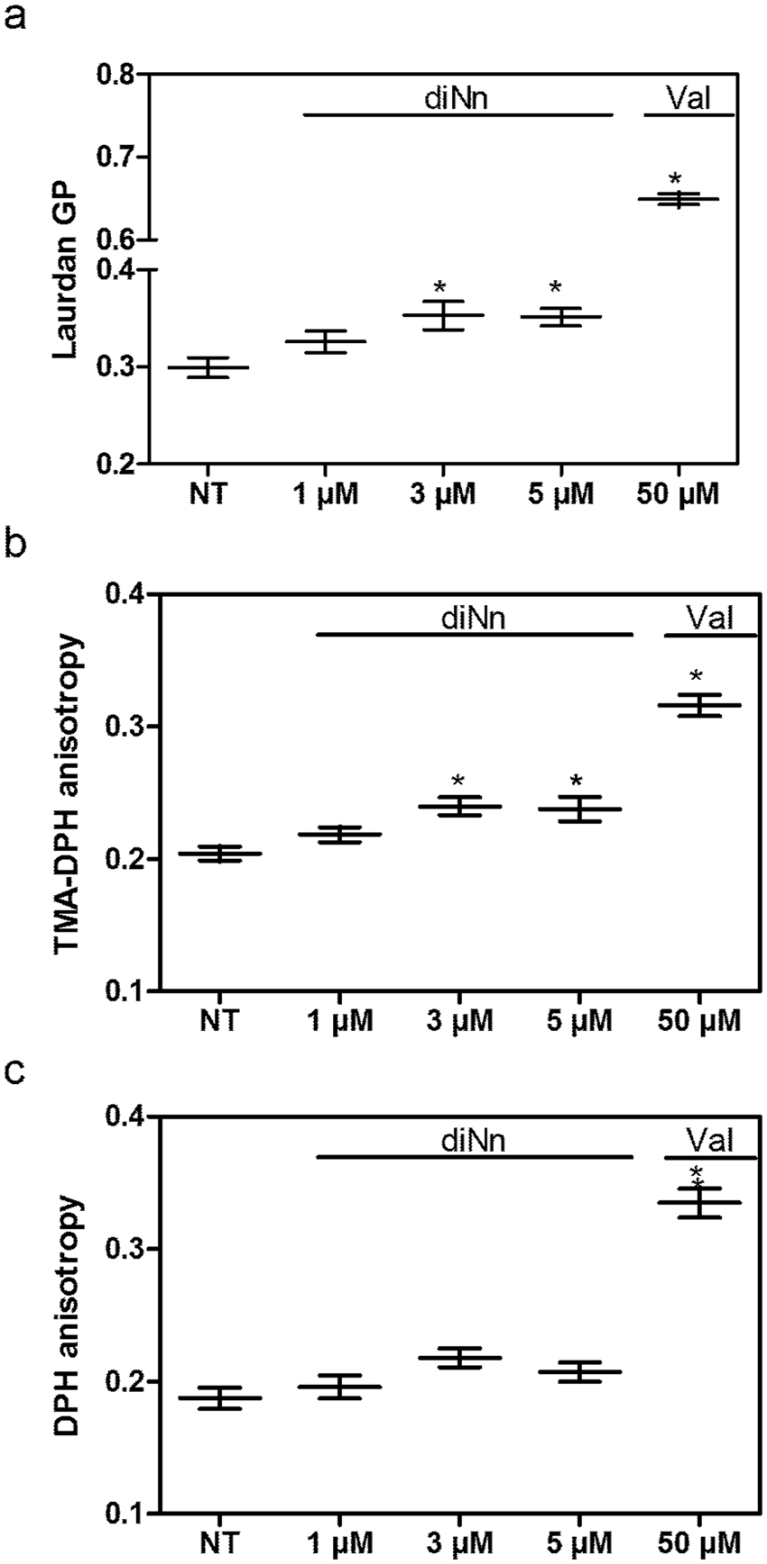
diNn decreases bacterial membrane fluidity/ hydration. Bacterial cells were incubated for 10 mins at 37 °C in the presence of diNn before analysis. (**a**) General polarization (GP) of Laurdan, (**b**) TMA-DPH and (**c**) DPH anisotropy in *P*. *aeruginosa* non treated (NT) cells or treated (T) with different concentrations of diNn. Valinomycin was used at 50 µM as a positive control. *p < 0.05 (N = 3, error bar represent SEM). Valinomycin was used at 50 µM as a positive control. *p < 0.05 (N = 3, error bar represent SEM).

All these features i.e. changes in lipid organization and lipid phases in membrane, and Zeta potential increase, might affect cell membrane fluidity / hydration. Laurdan, a polarity sensitive probe was used to detect changes in the general membrane fluidity/hydration. When the polarity of the lipid bilayer changes, a shift in Laurdan emission spectrum is detected and quantified by the general polarization (GP)^35^. When compared to non-treated bacterial cells, an increase in the GP was observed at concentrations of diNn ranging from 3 µM to 5 µM (Fig. 4a) indicating a decrease in membrane fluidity/hydration. We can speculate that electrostatic interactions between negative charges of cardiolipin and positive charges of diNn induce exclusion of water molecules from lipid head groups of the aqueous interphase which constitutes 40% of the total membrane thickness^36^. In fact, cardiolipin could form HII phase in the presence of diNn since membrane charge and cardiolipin phases (lamellar/ non lamellar) behavior are in wide correlation. HII phase is thermodynamically enhanced when surface charge increases and hydration decreases^37^. Interestingly, reports suggest that hydration changes of the lipid head groups are highly correlated to headgroup’s orientation, dipole potential and mobility through modulation of lipid-lipid and lipid-water interaction, which, in turn, affect lipid packing and dynamics in membrane and microdomains^34^.

Under the scope of better understanding fluidity changes and the extent of diNn insertion into the lipid bilayer, fluorescence anisotropy of 1-[4-(trimethylamino)phenyl]-6-phenyl-1,3,5-hexatriene (TMA-DPH) and 1,6-diphenyl-1,3,5-hexatriene (DPH) were assessed. TMA-DPH is anchored at the membrane aqueous interface while DPH penetrates the hydrophobic core of the membrane^38, 39^. Therefore, knowing the preferential location of each probe, slight changes in their polarization/anisotropy indicate fluidity discrepancies of the membrane^40^. An increase in TMA-DPH anisotropy was observed at concentrations ranging from 3 µM to 5 µM (Fig. 4b) whereas fluorescence anisotropy of DPH remained constant in the presence of diNn (Fig. 4c) with values comparable to those obtained with Baysse *et al*.^41^. Therefore, diNn inserts at the interfacial region of the membrane by adsorption to or penetration within the first layer. Although, the displacement of water from membrane interphase is accompanied with a decrease of the effective size of lipid head groups leading to membrane compression^42^, our previous results revealed that interaction of diNn with CL resulted in an increase in the lipid mean molecular area and a decrease in the fluidity of the membrane interphase. Thus, we can speculate that this increase of the lipid molecular area is the result of diNn insertion into the membrane.

## Targeting microdomains induces bacterial membrane permeabilization and morphological alterations

**Figure 5:**
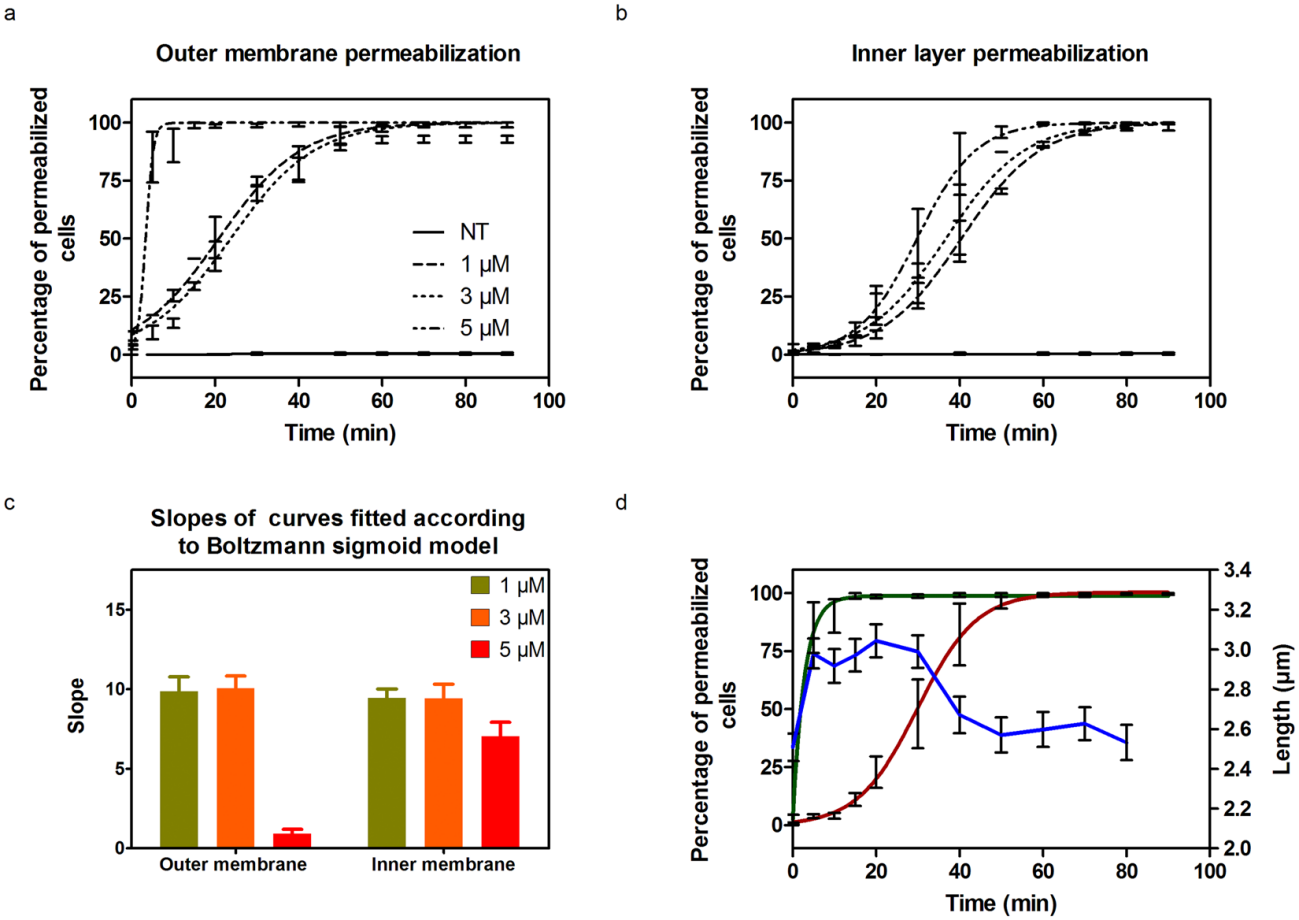
diNn induces outer and inner membrane permeabilization in addition to a decrease of bacterial cell length. Time lapse studies were conducted for 5 hours at 37 °C in CaMHB agarose pad supplemented with NPN, PI, and diNn when needed. Fluorescence and wide field images were analyzed in order to evaluate outer and inner membrane permeabilization and length of the cells. An increase in fluorescence indicates membrane permeabilization. Kinetics of the (**a**) outer and (**b**) inner bacterial membrane permeabilization in the presence of increasing concentrations of diNn. (**c**) Slopes of the sigmoidal curves fitting of outer and inner bacterial membrane permeabilization. The rate of permeabilization process is inversely proportional to the slope. According to one way ANOVA’s test and Tukeys comparison test, outer membrane permeabilization at 5 µM was significantly different (p < 0.001) than permeabilization at 1 µM and 3 µM whereas no statistically significant difference was observed in inner membrane permeabilization between the three concentrations (N = 3, values are mean ± SEM). (**d**) Outer (green) and inner (red) membrane permeabilization and bacterial length (blue) evaluation as a function of time and in the presence of diNn at 5 µM (N = 3).

Insertion of diNn within lipid bilayers could result in membrane permeabilization due to phase boundaries defects^19, 42^. Since membrane integrity plays a critical role for bacterial growth and survival^43, 44^, we monitored the effect of diNn on bacterial outer and inner membrane permeabilization. Time lapse studies of *P*. *aeruginosa*, by epifluorescence and wide field microscopy, were conducted in the presence of diNn and two fluorescent probes: 1-*N*-phenylnaphtylamine (NPN) and propidium iodure (PI) detecting outer and inner membrane permeabilization, respectively. When the outer membrane is permeabilized, NPN accumulates in the membrane’s hydrophobic core^45^ and when inner membrane permeabilization occurs, PI passes through the cell membrane and binds to nucleic acids^46, 47^. In both cases an increase in fluorescence is observed. The percentage of NPN and PI fluorescent cells, for non-treated cells or in the presence of different concentrations of diNn, was plotted as a function of time and concentrations of diNn (Fig. 5a,b). Outer membrane permeabilization occurred immediately in the presence of diNn whereas inner membrane permeabilization was triggered about 10 min after outer membrane permeabilization. Both of the curve sets were fitted using the Boltzmann sigmoidal function in order to determine the slope of the curves corresponding to the permeabilization rate (Fig. 5c). The slope relative to outer membrane permeabilization was markedly decreased at 5 µM. The effect was slighter for inner membrane permeabilization. In parallel, possible effects on bacterial shape were investigated. In Fig. 5d, mean bacterial length and outer and inner membrane permeabilization at 5 µM of diNn are plotted as a function of time. Cells were elongating for the first 5 mins of incubation until outer membrane permeabilization was triggered. This was followed by a length stabilization followed by an obvious decrease in the bacterial length after 30 min of incubation where more than 50% of the cells had outer and inner membranes permeabilized.

**Figure 6:**
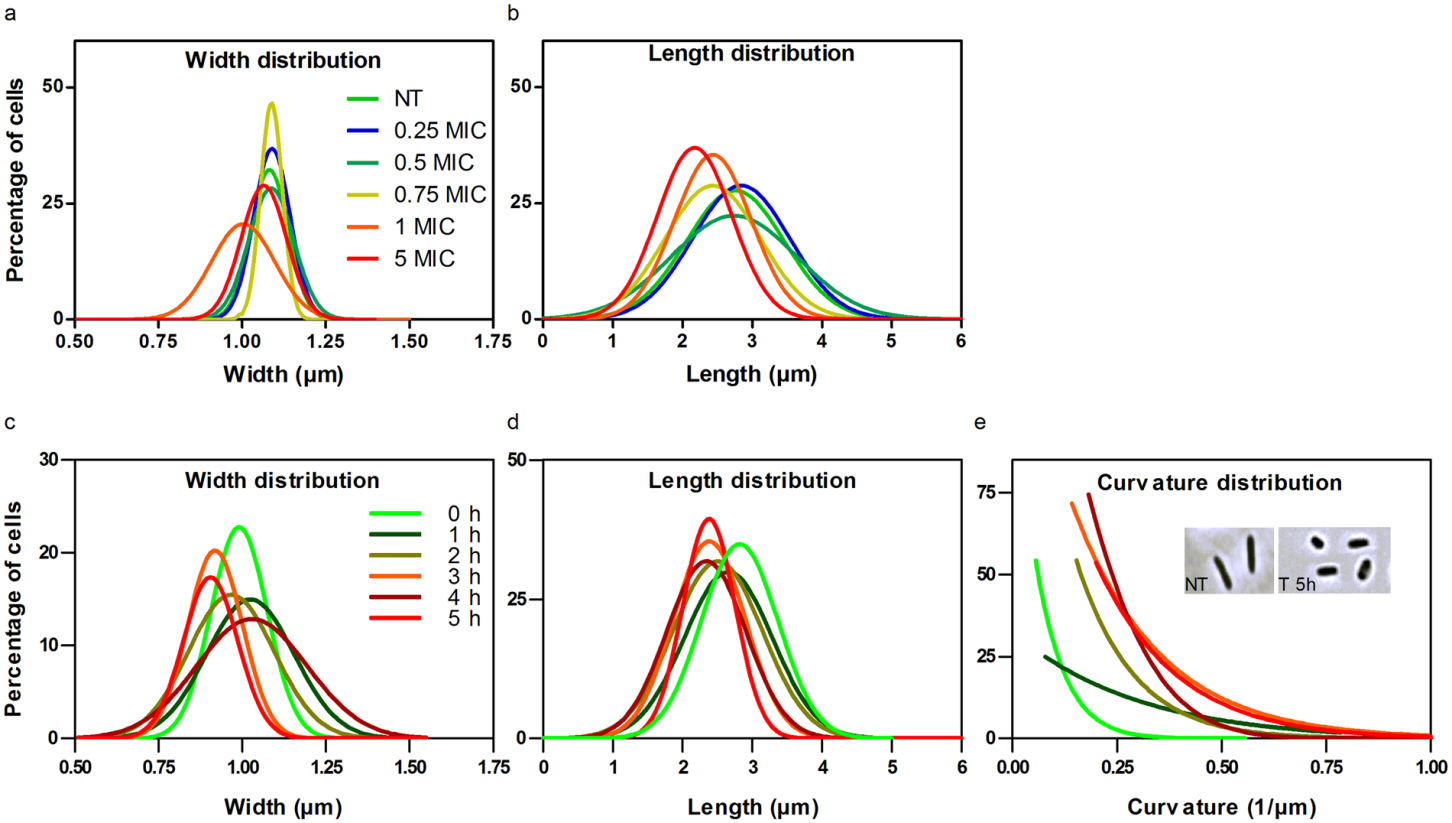
diNn alters bacterial length and curvature of *P*. *aeruginosa* cells in a time dependent manner. Time lapse studies were conducted for 5 hours at 37 °C in CaMHB agarose pad supplemented with the neamine derivative (**a**,**b**). Wide field images were analyzed and the (**a**) length and (**b**) width distribution of the cells were calculated. *P aeruginosa* cells were incubated in the presence (T) or absence (NT) of diNn at its MIC; the cells were imaged and analyzed as a function of time (**c**,**d**). (**c**) Distribution of the bacterial cells width, (**d**) length, and (**e**) curvature up to 5 hours of incubation; insert shows wide field microscopy images of non-treated (left) or treated (right) bacterial cells after 5 hours of incubation. (N ≥ 3)

A more extensive study was then conducted on bacterial shape (length, width, and curvature) through time lapse experiments as well as time- and dose- effects. To facilitate the interplay between basic sciences and clinics, concentrations will be further expressed in minimal inhibitory concentration MIC ratios where the MIC of diNn on *P*. *aeruginosa* is about 7 µM^16^. The effect of diNn was compared to gentamicin, a conventional aminoglycoside antibiotic, colistin, a drug targeting bacterial outer and inner membranes, and neamine being the core component of diNn. These drugs were embedded in agarose pad prepared with the culture medium at their respective MICs. Analysis of bacterial cell length and width distribution concluded that only diNn induces a significant decrease of the bacterial length. Colistin, gentamicin, and neamine had no significant effect on cells width and length distributions (SI, Fig. S2). In some cases (width with diNn and neamine), the distribution showed no statistical significant alterations when compared to control cells although a more dispersed distribution was observed. When testing different concentrations of diNn, no statistically significant effect was observed on cells width (Fig. 6a) whereas a clear dose effect on the bacterial length was detected starting at 0.75 MIC (Fig. 6a). Analysis of morphological effects and effect of diNn on bacterial membrane curvature was conducted in liquid medium. The width of cells was stable throughout 5 hours of incubation (Fig. 6c), and their length decrease was time dependent with bimodal behaviors (Fig. 6d). A severe increment in cells curvature was also detected (Fig. 6e). For instance, after 3 hours of incubation, curvature distribution was shifted 3 folds to higher values (*SI*, Fig. S3) and cells lost their initial rod shape (Fig. 6e, insert).

**Figure 7:**
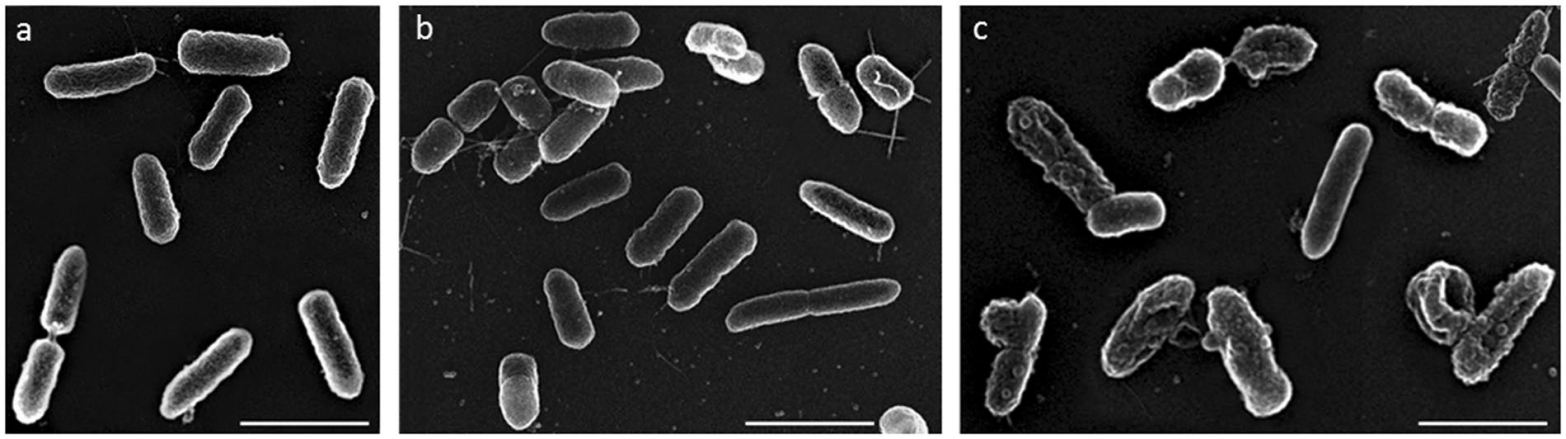
diNn induces defects on *P*. *aeruginosa* cells morphology. *P*. *aeruginosa* were incubated in the presence of diNn at 1 and 5 µM for 1 hour before sample preparation. (**a**) Scanning electron microscopy of control and (**b**,**c**) cells previously treated with diNn at 1 and 5 µM, respectively. Scale bars correspond to 2 µm. Damage in the cell membrane can be seen when compared with the control sample.

**Figure 8:**
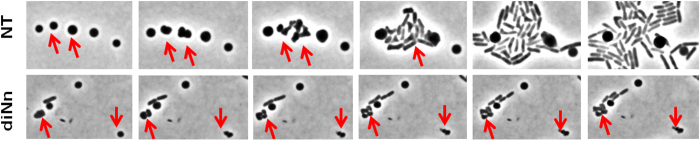
diNn inhibits the regeneration of rod-shaped cells from L-spheroplasts. The non-treated (NT) spheroplasts which divided (red arrows) regenerated their rod shape after 3 divisions. In the presence of diNn, spheroplasts lost totally their shape and their division potential after 2 cycles. Representative images of two independent experiments are shown.

In order to study in more details the observed effect on macroscopic cell curvature, scanning electron microscopy was conducted on non-treated or treated *P*. *aeruginosa* cells with different concentrations of diNn in order to detect any microscopic curvature defects (*SI*, *Methods*). Figure 7 shows a local increase in bacterial cells curvature and a decrease in bacterial length (Fig. 7b versus Fig. 7a). At 5 µM (around 0.75 MIC) (Fig. 7c), diNn induced severe morphological defects characterized by membrane blebbing (regions with high membrane curvature), loss of rod morphology, increase in overall membrane curvature, and depressions on the surface. The appearance of these highly curved microscopic regions could be the consequence of cardiolipin redistribution, fluidity changes and enhancement in lipid phase separation. The latter can be explained either by changes in (i) lipids proportion in the outer layer as compared to the inner layer of the membrane and/or (ii) CL-interacting proteins implicated in cell shape regulation. In the first instance, based on results from spheroplasts (Fig. 8), we can speculate that the insertion of diNn (inverted cone shape)^16^ into the membrane together with its high affinity to cardiolipin could induce cardiolipin enrichment into the membrane’s outer layer, leading to the increase in membrane curvature. In accordance with our previous study where diNn induced lipid mixing in liposomes^17^, transfer of cardiolipin from bacterial inner membrane to the outer membrane^48^ could explain the effect on curvature. In the second instance, cardiolipin could interact with membrane proteins and regulate the position and function of proteins in bacterial cells. We can speculate that when diNn binds to cardiolipin via electrostatic and hydrophobic interactions, cardiolipin-protein interactions are affected, leading to alteration of proteins distribution, function and dynamics. Among them, those involved in cell division regulation (MinD, e.g.)^8, 9^, cell division (FtsZ, FtsA)^3^ and cell shape (MreB) have been described. Based on the decrease of fluidity induced by diNn and by the fact MreB, an actin homolog protein associated with lipids that are in fluid, liquid-disordered state^49^, we questioned the potential role of this cardiolipin-interacting protein in the effect afforded by diNn. MreB is a curvature dependent protein^50^ that interacts with bacterial membranes via its N-terminal amphipathic helix^51, 52^ MreB localization determines the basic rod shape, whereas MreB dynamics promotes robust rod-like morphogenesis and prevents these local defects from being amplified^53, 54^. By analyzing the localization and dynamics of mCherry labelled MreB in *P*. *aeruginosa*, time-lapse microscopy with control cells or in the presence of diNn (*Supplementary video*, SM. 1, SM. 2) revealed two important features. First, diNn disrupted mCherry-MreB localization which became diffuse. Second, MreB did not move but was rather statically located underneath the cell membrane in strong contrast to control cells. Interestingly, this effect on MreB could be also related to potential defects in cell wall suggested by the outer and inner membranes permeabilization induced by diNn as well an effect on membrane depolarization^17^, a critical parameter for MreB optimal efficiency. In addition to these effects, we also evidenced defects in cell wall material synthesis since we observed the inability of *P*. *aeruginosa* L-spherosplasts to regenerate their initial rod shape in the presence of diNn (Fig. 8).

## Disruption of cellular functions upon cardiolipin clustering and microdomains defects

**Figure 9:**
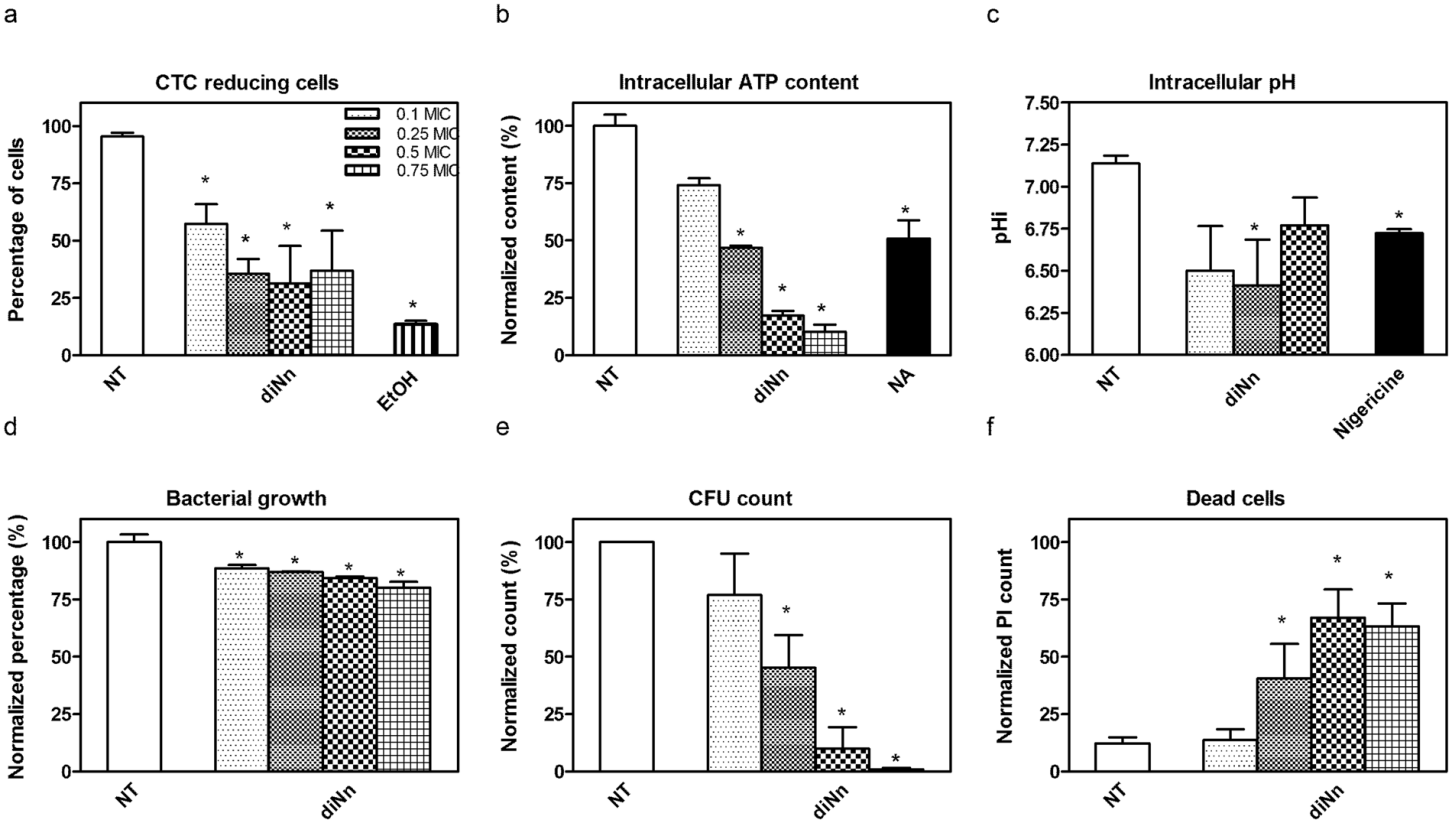
diNn inhibits the respiratory chain in *P*. *aeruginosa* cells leading a dose-dependently decrease of the bacterial growth rate, CFU count, and cell death. Effect of diNn after 10 min of incubation on (**a**) CTC redox chain, (**b**) intracellular ATPi, (**c**) intracellular pHi, (**d**) bacterial growth rate, (**e**) CFU, and (**f**) dead cells counts. Results in b, d and c are expressed in percentage when compared to the control condition. (N ≥ 3, Values are mean ± SEM). *P < 0.05; in comparison to untreated control cells.

If cardiolipin plays a key role for maintaining bacterial shape, this lipid also influences the organization and activity of proteins involved in bacterial oxidative phosphorylation. Cardiolipin bound between complexes subunits or at monomer interfaces of oligomers and acts as a flexible amphipathic linkage between proteins and membrane^7, 8^. This tight interaction stabilizes protein complexes and regulates their function^9^. In order to have insight on the effect of diNn on bacterial functionalities, we analyzed the activity of the redox chain, the intracellular ﻿ATP content (ATPi) and pH (pHi), the bacterial growth rate, cells viability expressed in colony forming unit (CFU) and cells mortality (Fig. 9). These parameters were tested at sub MICs concentrations (from 0.1 to 0.75 X MIC). A clear inhibition of the redox chain was observed accompanied with a decrease in the ATPi content and pHi. Moreover, the growth rate of *P*. *aeruginosa* decreased in the presence of increasing concentrations of diNn. A severe decrease in CFU count was also detected accompaned with an increase in cell mortality. This effect was not observed for colistin, gentamicin and neamine (*SI*, Fig. S4). Again, effect on cardiolipin microdomains could explain these functional defects in the respiratory chain producing a decrease in ATPi content and pHi. In *E*. *coli*, cardiolipin is tightly bound to proteins of the respiratory chain including succinate deshydrogenase^7, 11, 12^. For instance, succinate deshydrogenase transmembrane domains are stabilized by hydrogen bonds with cardiolipin and phosphatidylethanolamine. Cardiolipin is also involved in the generation of Δψ by entrapping protons within head groups. Finally, cardiolipin is the most important anionic phospholipid which plays a role in DNA replication by regulating DnaA^9^. Hence, when diNn interacts with cardiolipin headgroup and decreases membrane hydration, it could hinder the interaction between cardiolipin and succinate deshydrogenase leading to proteins dysfunction/misfolding, proton entrapping through cardiolipin and DnaA regulation. If all of these data point out the key role of cardiolipin in membrane and membrane protein structure and functions, further studies are required to understand the molecular mechanisms involved. One appealing approach would be to modify the cardiolipin content by using molecular biology or biochemical approaches, e.g. Regarding the molecular biology approach, suppressing the cardiolipin synthesis in *P*. *aeruginosa* is highly tricky and literature only refers to study performed on *S*. *aureus* ^55, 56^, *B*. *subtilis* and *B*. *firmus* ^57^, and *P*. *putida* ^22, 58^. From these studies, it clearly appears that synthesis of cardiolipin is complex and is highly dependent of several isoforms of cardiolipin synthase, which all of them contribute to cardiolipin synthesis in stationary phase^59^. As an example, in *E coli*, only bacteria with deletions of all three of the genes encoding cardiolipin synthases, ΔclsABC, completely lack cardiolipin^59^. Moreover, discovery of a bifunctional cardiolipin / phosphatidyl ethanolamine synthase in bacteria has to be taken into account as described for *E*. *coli* ^59^ and *X*. *campestris* ^60^. Another common approach to modify the cardiolipin content is to culture bacteria under high salt concentrations or to add exogeneous cardiolipin during the bacterial growth. Increase in cardiolipin content under conditions of high salinity has been largely reported both in Gram-positive bacteria including *B*. *subtilis* ^61, 62^ and *S*. *aureus* ^63^ and in Gram-negative bacteria including *R*. *sphaeroides* ^64^, *C*. *salexigens* ^65^, and *E*. *coli* ^66^. Unfortunately these approaches also show drawbacks since increase in NaCl concentrations could affect the behavior of free and/or bound diNn to membrane components favoring aggregation in solution and/or proximity of bound diNn in the membrane, respectively. When cardiolipin was exogeneously added, more complex processes than it seems at first sight could appear, including (i) potential effect on cardiolipin synthases, (ii) potential displacement of diNn from its primarily binding to LPS^16^, (iii) ration of cardiolipin located within the outer and the inner membranes, in relation with lipid transfer but also with the activity of proteins involved in cardiolipin transfer from the inner membrane to the outer membrane like PbgA in Gram-negative bacteria^48^ and lastly, (iv) equilibrium between the interaction of cardiolipin with the diNn and with proteins depending upon cardiolipin and involved in bacterial shape and division.

## Conclusions

Through targeting cardiolipin in membrane models, lipid reorganization occurs together with an enhancement of lipid phase separation and cardiolipin clustering. In bacterial cells, cardiolipin microdomains disassemble into cardiolipin clusters coupled with a decrease in membrane fluidity and phase boundaries modifications leading to membrane permeabilization. As a result, bacterial cells undergo severe morphological defects including an increase of curvature and a decrease in length. The curvature increase can be due, in part, to the insertion of diNn into the membrane and the enrichment of cardiolipin in the membrane outer layer favored by diNn cooperative binding to cardiolipin. Structure/function of proteins implicated in cell shape regulation and biological processes, and interacting directly with cardiolipin or curvature dependent proteins can be hindered as a result of diNn interaction with cardiolipin. By targeting cardiolipin microdomains in bacteria, microdomain assembly would be altered leading to dysfunction of proteins involved in cell division, cell shape and oxidative phosphorylation.

## Experimental procedure

### Time lapse of *P*. *aeruginosa* and dimension analysis

Aliquots of mid-log cultures were deposited on a 1% MHB-CA agar pad supplemented or not with antibiotics at different concentration. NPN and PI were also added to the agarose pad. Cell imaging was done every 10 mins using Axioskop microscope (Zeiss) equipped with Orca Flash 4.0 camera (Hamamatsu). For dimensions studies, cells length, width and curvature were analyzed using the Matlab-based open source software Microbe Tracker^67^, and Oufti^68^.

### TF-CL and NAO labelled bacteria

Fresh mid-log cultures washed in TrisHCl 30 mM (pH 7.8) were labelled with TopFluor®-cardiolipin (TF-CL) (Avanti Polar Lipids, Alabaster, AL). diNn was added to TF-CL labelled cells or to unlabelled cells and incubation was done for 20 min at 37 °C. NAO (A7847; Sigma-Aldrich) was used to visualize CL domains in non labelled cells^20^ followed by labeling DNA with DAPI (D9564; Sigma-Aldrich). The fluorescent dyes were excited using a mercury lamp (EXFO Life Sciences) and the appropriate filters (DAPI: λex = 353 nm/λem = 465 nm; NAO: λex = 488 nm/λem = 639 nm; TF-CL: λex = 495 nm/ λem = 520 nm).

### Bacterial cytoplasmic membrane fluidity changes assessed by Laurdan, TMA-DPH and DPH

Laurdan was used to detect membrane fluidity/hydration changes through a shift in its emission spectrum. The steady state fluorescence parameter known as excitation generalized polarization (GP) was calculated. Anisotropies of TMA-DPH and DPH reflecting membrane fluidity at the aqueous membrane interface or the hydrophobic core respectively, were determined. Valinomycin was used as a positive control (*SI appendix*, *SI* methods).

### mCherry-MreB imaging

*P*. *aeruginosa* producing mCherry-MreB^53^ was kindly supplied by Pr. Gitai (Princeton University). Overnight bacterial cultures were subcultured into LB broth and incubated for 3 h at 37 °C. To monitor the distribution and motion of MreB in living cells, 4 µL of the bacterial cultures were spotted on LB agarose pad supplemented or not with diNn at 4 µg/mL. The slides were incubated at 37 °C for 10 min before collecting phase contrast and fluorescence images every 30 s for 5 min.

### L-spheroplasts preparation and incubation and shape recovery assay

L-spheroplasts were prepared according to Ranjit *et al*.^1^. Briefly, *P*. *aeruginosa*’s mid-log cells were washed twice in PBS, and the final pellet was resuspended in buffer (PBS, 0.5 M sucrose, and 20 µg/mL lysozyme). After an incubation of 10 min at 37 °C, an equal volume of the same buffer was added to the suspension, which was incubated at 37 °C for an additional 10 min followed by a further incubation for 15 min. Whole cells and spheroplasts were washed in sucrose recovery medium (2% tryptone, 0.5% yeast extract, 10 mM NaCl, 2.5 mM KCl, 10 mM MgCl_2_, 10 mM MgSO_4_, 20 mM glucose, 0.23 M sucrose, pH 7). An aliquot was placed onto sucrose recovery soft agarose (0.7%) pad supplemented or not with diNn and a time lapse experiment was conducted as described above.

## Growth rate

Growth rate of *P*. *aeruginosa* was studied in 24 well culture plates in the presence of diNn, colistin, gentamicin, and neamine at different concentrations. The plates were incubated in SpectraMax®M3 548 Microplate Reader at 37 °C and the optical density was followed for 12 hours in order to analyze the growth rate (*SI appendix*, *SI methods*).

## Cellular functions: 5-cyano-2, 3-ditolyl tetrazolium chloride (CTC) redox assay, ATP,  propidium iodide, and CFU counts

The effect of diNn on cells was analyzed after 10 min of incubation. We used BacLight RedoxSensor CTC vitality kit (Molecular Probes, Eugene, OR) to visualize bacterial respiration, propidium iodide (PI) for labelling dead cells, ATP Determination Kit (Life technologies, Grand Island, NY, USA) for quantifying intracelluler ATP, cFSE probe (Life technologies, Grand Island, NY, USA) for the evaluation of the intracellular pH, and CFU counts (*SI appendix*, *SI methods*).

## Electronic supplementary material

Supplementary Information

Supplementary Movie 1

Supplementary Movie 2
