# Supplementary material for: Targeting Bacterial Cardiolipin Enriched Microdomains: An Antimicrobial Strategy Used by Amphiphilic Aminoglycoside Antibiotics
Source: Sci Rep. 2017 Sep 6;7:10697. doi: 10.1038/s41598-017-10543-3 (PMC5587548; doi:10.1038/s41598-017-10543-3)
Supplement: Supplementary file 1 — Supplementary Information [file 41598_2017_10543_MOESM1_ESM.pdf]

SI APPENDIX

Targeting Bacterial Cardiolipin Enriched Microdomains: An Antimicrobial Strategy Used by Amphiphilic Aminoglycoside Antibiotics.

Micheline El Khoury, Jitendriya Swain, Guillaume Sautrey, Louis Zimmermann, Patrick Van der Smissen, Jean-Luc Decout, Marie-Paule Mingeot-Leclercq

Chemical structures of 3',6 dinonyl neamine, lipids, and fluorescent probes.

- 3',6-dinonyl neamine

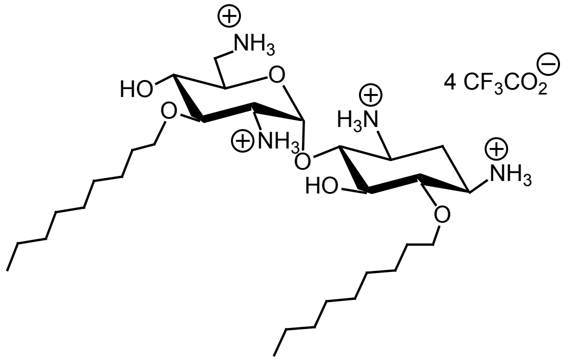

- Cardiolipin: R_1, R_2, R_3 and R_4= fatty acid hydrophobic chains

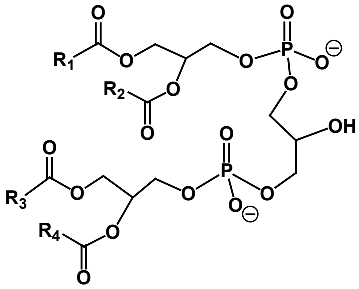

- Phosphatidylglycerol: R_1 and R_2= fatty acid hydrophobic chains

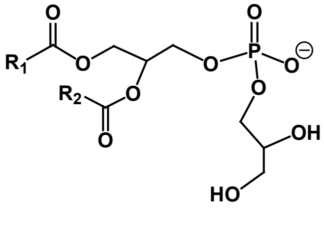

- Phosphatidylethanolamine: R_1 and R_2= fatty acid hydrophobic chains

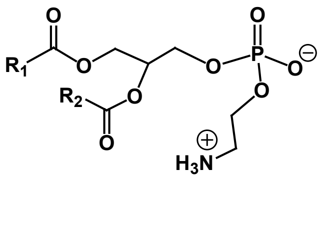

- TF-CL: Top-Fluor cardiolipin

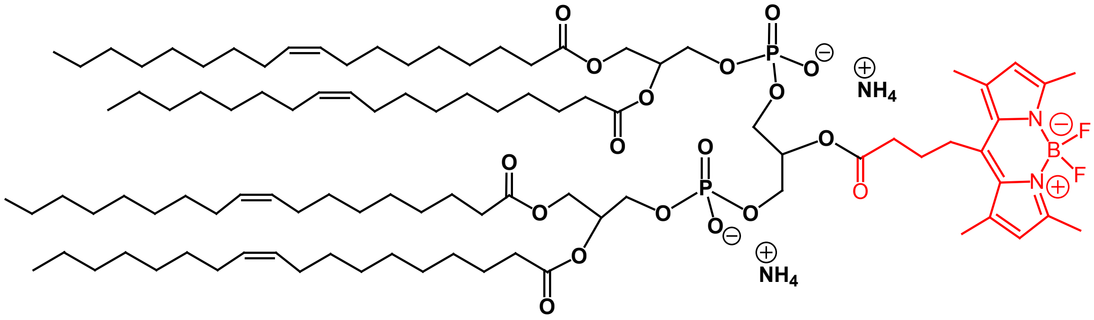

- TR-PE: Texas Red phosphatitylethanolamine

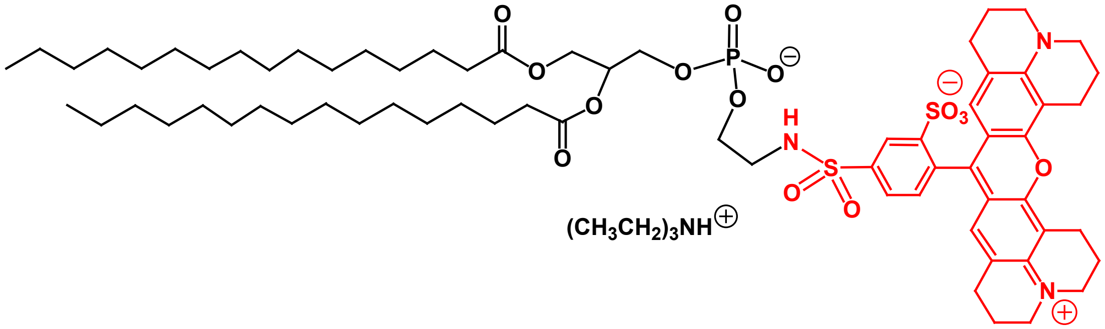

- NBD-PE: *N*-(7-nitrobenz-2-oxa-1,3-diazol-4-yl)-1,2-dihexadecanoyl-*sn*-glycero-3-phosphoethanolamine

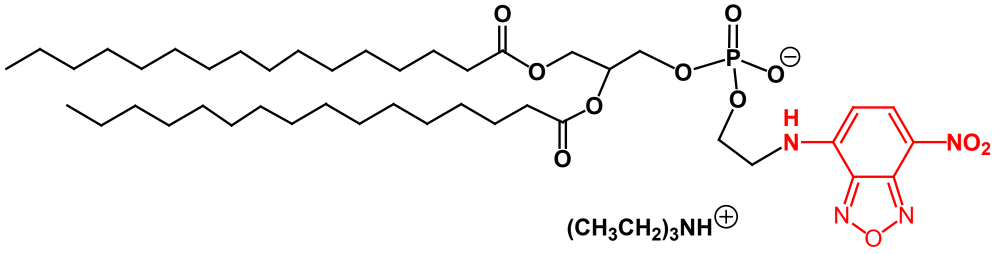

- NAO: 10-*N*-nonyl acridine orange

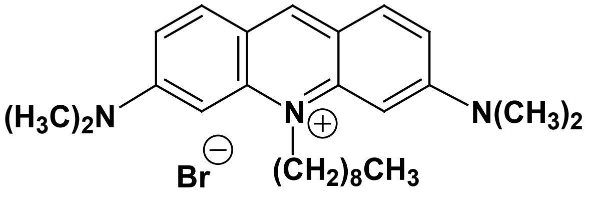

- TMA-DPH: Trimethylammonium 1,6-diphenyl-1,3,5-hexatriene

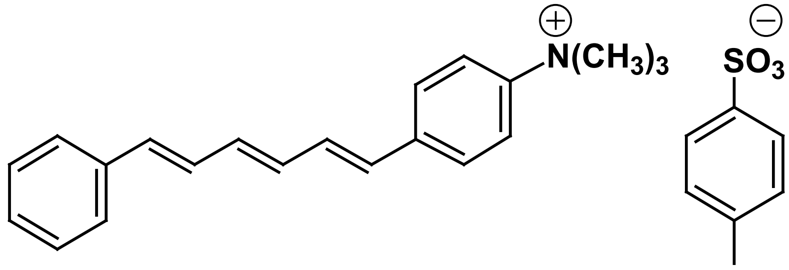

- DPH: 1,6-diphenyl-1,3,5-hexatriene

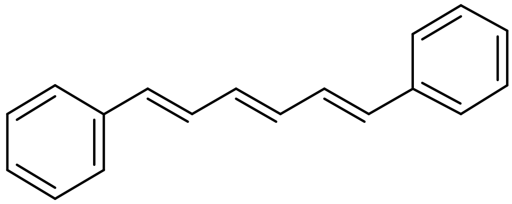

I. SI METHODS

Bacterial strain and growth conditions

Two *P. aeruginosa* strains were used: ATCC27853 and PAO1 producing mCherry labelled MreB (1). Trypticase soy agar (TSA) medium was used to grow *P. aeruginosa* ATCC 27853 overnight at 37 °C. One colony was suspended in Cation Adjusted –Müller Hinton Broth (CaMHB) and incubated overnight at 37 °C on a rotary shaker (130 rpm). The bacterial suspension was diluted 100 fold in CaMHB and incubated (130 rpm; 37 °C; 4 h) until it reached the mid logarithmic (mid-log) phase (OD620 ~ 0.4–0.5).

Giant unilamellar vesicle (GUV) preparation

GUVs were prepared by electroformation method (2). Lipids were mixed in respective molar ratios of PE/PG/CL (60/21/11) (3, 4). GUVs were labeled either with TR-PE (0.1 mol%) or with TR-PE (0.1 mol%) and TF-CL (0.2 mol%) . The lipid mixture was spread (2.5 µg/cm2) on a glass slide coated with indium tin oxide (ITO) and dried for 15 min under vacuum. To build the electroformation chamber, the first slide was covered with a second ITO-coated slide spaced with PDMS containing 5 % fumed silica. The electroformation chamber was then filled with buffer (2.5 mM TrisHCl; pH 7.4; and 0.2 M saccharose) and closed with PDMS. The GUVs were formed by applying a sinusoidal alternating current of 500 Hz and 3.25 V for 2 h at 45 °C. GUVs were detached from the ITO-coated slide and collected. 50 µL of GUVs were

diluted in glucose buffer (2.5 mM TrisHCl; pH 7.4; and 0.2 M glucose) with and without addition of diNn and introduced into an Ibidi chamber.

Molecular modeling

The conformation of the molecules (Neamine, 3´,6-diNonyl Neamine (diNn), and cardiolipin with palmitoyl and oleyl alkyl chains) was calculated with the structure tree procedure as described elsewhere (5). We used the Hypermatrix docking procedure to study the interaction between diNn and the lipid molecules (as described (5-9). To perform this calculation, the diNn molecule is positioned and fixed at the center of the system, oriented at the hydrophobic (pho)/hydrophilic (phi) interface (10). The lipid molecule is also oriented at the pho/phi interface; through rotations and translations, more than 107 positions of the lipid around the central molecule are calculated. The energy values are stored in a matrix together with the coordinates of all assemblies and classified according to decreasing values. The most stable match is used for the position of the first lipid. The position of the second lipid is then defined as the next most energetically favorable orientation stored in the matrix, taking into account steric and energetic constraints resulting from the presence of the first lipid molecule. The process ends when the central molecule is completely surrounded by lipids. This method takes into account the lipid/water interface through linear variations of the dielectric constant Ɛ ranging from 3 (above the interface) to 30 (below the interface). An empirical equation for the hydrophobic energy is added in the force field (as described in (11)).

The mean area in the complex occupied by either a lipid or by diNn was estimated through x-y plane projections using a grid of 1 Å square. The mean calculated interfacial area corresponds to the area measured experimentally with a Langmuir monolayer trough under weak compression conditions (9).

To calculate the insertions of Neamine and diNn into an implicit simplified bilayer, we used the integral membrane protein and lipid association (IMPALA) method described elsewhere (12). In sum, this method simulates the insertion of any molecule into a bilayer by adding energy restraint functions to the usual energy description of molecules. The lipid bilayer is defined by C(z), which represents an empirical function describing membrane properties. This function is constant in the membrane plane (x- and y-axis), but varies along the bilayer thickness (z-axis). Two restraints simulate the membrane: one is the bilayer hydrophobicity (Epho), the other is the lipid perturbation (Elip). All equations have been described elsewhere (12). Calculations were performed on a Linux station (bi-xeon quad core) using in-house Z-ultime software.

Bacterial cytoplasmic membrane fluidity changes assessed by Laurdan, TMA-DPH and DPH

Laurdan was used to detect membrane fluidity/hydration changes through a shift in its emission spectrum. The steady state fluorescence parameter known as excitation generalized polarization GP quantitatively relates these spectral changes. TMA-DPH and DPH were used to study membrane anisotropy. TMA-DPH is anchored at the aqueous membrane interface while DPH is an extremely hydrophobic probe that penetrates into the hydrophobic core of the membrane. Therefore, TMA-DPH and DPH anisotropies reflect membrane fluidity at the aqueous membrane interface or the hydrophobic core respectively

Mid log cells were harvested and washed either in Laurdan prewarmed buffer [50mM Na_2HPO_4/NaH_2PO_4 pH 7.4, 0.1 % glucose and 150 mM NaCl] or prewarmed buffer for anisotropy measurements [50 mM Na_2HPO_4, 50 mM KH $_2PO_4$, 150 mM NaCl, 60 mM $(NH_4)_2SO_4$, pH 7.4] to an optical density of 0.05 at 600nm.

For experiments with Laurdan, cells were incubated with diNn for 10 min at 37 °C, washed with Laurdan prewarmed buffer and labelled with 2 µM Laurdan for 1 h at 37°C. Cells were then washed three times in prewarmed buffer. Laurdan fluorescence emissions were measured at 440 and 490 nm upon excitation at 350 nm, using a Perkin Elmer LS-55 spectrofluorimeter. GP was calculated applying the formula:

$$GP = \frac{I1 - I2}{I1 + I2}$$

where *I*1 is the fluorescence intensity at 440 nm and *I*2 is the fluorescence intensity 490 nm.

For anisotropy experiments, 2 µM of TMA-DPH or DPH were added to bacterial suspension and incubated at 37 °C for 10 min (TMA-DPH) or 45 min (DPH).

The cuvette holder temperature was set at 37 °C. The readings were taken after 10 min of incubation. The polarization values (r) of the samples were calculated by the fluorescence data manager program using the Jablonski equation:

$$r = \frac{Ivv - GIvh}{Ivv + 2GIvh}$$

where *Ivv* and *Ivh* are the vertical and horizontal fluorescence intensities, respectively, of the vertical polarization of the excitation light beam. The factor $G = Ihv/1hh$ (grating correction factor) corrects the polarizing effects of the monochromator. The excitation wavelengths were 340 nm (TMA-DPH) and 348 nm (DPH), and the fluorescence emission was measured at 430 nm for TMA-DPH and 426 nm for DPH.

In all experiments, valinomycin was used as a positive control (13).

Liposome preparation and zeta potential measurement

Phospholipids PE/PG/CL (5 mg/mL in $CHCl_3/CH_3OH$ 2:1 v/v) were mixed in the desired molar ratio, namely, 60/21/11. MLVs were prepared according to the freeze-thawing method in an aqueous buffer (10 mM TrisHCl; pH 7.4). LUVs were then obtained with 10 successive extrusions of MLVs through 2 polycarbonate membranes (pore size =50 nm; Avanti Polar lipids) using a mini extruder (Avanti Polar lipids). Dynamic light scattering measurements were done using a Nanosizer ZS (Malvern Instruments) to control the size and polydispersity of the LUVs (2). The phospholipid concentration in LUVs suspension was determined with a Bartlett phosphate assay (14). LUVs were then diluted at 5 µM, incubated for 10 min with diNn at different concentrations, and the Zeta potential was determined by laser Doppler velocimetry using a Nanosizer ZS with a universal dip cell (Malvern Instruments).

Bright field microscopy of *P. aeruginosa* and dimension analysis

P. aeruginosa preculture was diluted 100-fold in fresh CaMHB and incubated at 37 °C in both the absence and the presence of diNn at its minimal inhibitory concentration MIC (4 µg/mL). Every hour, small aliquots were collected and observed using Axioskop microscope (Zeiss), Orca Flash 4.0 camera (Hamamatsu), and Zen 2012 software (Zeiss).

For time lapse experiments, bacteria's in midlog phase were deposited on a 1 % MHB-CA agar pad supplemented or not with the diNn, colistin (Col), Gentamicin (Gent) , and neamine (Nea) tested at different concentrations depending on their MICs against *P. aeruginosa* (1 µg/mL for Col and Gent, and > 128 µg/mL for Nea (15)). For outer and inner membrane permeabilization, NPN and PI were added to the agarose pad. Then, the agarose pads were covered, and sealed with VALAP according to (16).

For dimension (length, width, and curvature) and fluorescence studies cells were analyzed using the Matlab-based open source software Microbe Tracker (17), and Oufti (18).

NPN and PI fluorescence per cell were quantified, and cells with permeabilized outer and inner membranes were counted and plotted against the corresponding concentration of diNn. The curves were then modeled using the Boltzmann sigmoidal function given below:

$$Y = Yi + \frac{Yf - Yi}{e^{\frac{(v50-c)}{\gamma}}}$$

Were *Yi* and *Yf* are the minimum and maximum values respectively, *V50* corresponds to the inflection point, C corresponds to the concentration of diNn, and γ is the slope of the curve which is inversely proportional to *Y*.

Scanning electron microscopy

Non treated *P. aeruginosa* in mid-log phase or treated with diNn at different concentrations and incubation time were washed in phosphate buffered saline (0.1 M PBS, pH 7.4). The cells where then fixed for 30 min at room temperature in 4 % formaldehyde and 1 % glutaraldehyde in PBS. A suspension of bacterial cells was immobilized on poly-L-lysine coated coverslips for 10 min at room temperature. After washing in buffer – to remove the excess of free floating bacteria- the coverslips were incubated for a second fixation in 1 % glutaraldehyde in order to cross-link the already fixed bacteria on the poly-lysine.

We further post-fixed the samples in 1 % osmium tetroxide in cacodylate buffer for 2 h at 4 °C and washed extensively in water. Samples were then dehydrated in graded series of ethanol, critical point dried and coated with 10 nanometer of gold. Samples were observed in a CM12 Philips electron microscope at 80 kV with the secondary electron detector.

mCherry-MreB imaging

P. aeruginosa producing mCherry-MreB (1) was kindly supplied by Pr. Gitai (Princeton University). Overnight bacterial cultures were subcultured into LB broth and incubated for 3 h at 37°C. To monitor the distribution and motion of MreB in living cells, 4 µL of the bacterial cultures were spotted on LB agarose pad supplemented or not with diNn at 4 µg/mL. The slides were incubated at 37°C for 10 min before collecting phase contrast and fluorescence images every 30 s for 5 min.

L-spheroplasts preparation and incubation and shape recovery assay

L-spheroplasts were prepared according to Ranjit et al. 2013 (19). Briefly, *P. aeruginosa*'s mid-log cells were washed twice in PBS, and the final pellet was resuspended in buffer (PBS, 0.5 M sucrose, and 20 µg/mL lysozyme). After an incubation of 10 min at 37 °C, an equal volume of the same buffer was added to the suspension, which was incubated at 37 °C for an additional 10 min followed by a further incubation for 15 min. Whole cells and spheroplasts were washed in sucrose recovery medium (2 % tryptone, 0.5 % yeast extract, 10 mM NaCl, 2.5 mM KCl, 10 mM $MgCl_2$, 10 mM $MgSO_4$, 20 mM glucose, 0.23 M sucrose,

pH 7). An aliquot was placed onto sucrose recovery soft agarose (0.7 %) pad supplemented or not with diNn and a time lapse experiment was conducted as described above.

CTC redox assay, propidium iodide, and colony forming units CFU counts

The BacLight RedoxSensor 5-cyano-2, 3-ditolyl tetrazolium chloride (CTC) vitality kit (Molecular Probes, Eugene, OR) was used to visualize bacterial respiration as described previously (20). Briefly, cells in mid log were washed, resuspended in PBS at 10^6 CFU/mL, and incubated for 10 min at 37 °C in the presence of different compounds. Afterwards, cells were washed in PBS, stained in CTC solution (5 mM CTC, 20 mM glucose) , and counterstained in SYTO-24 (Molecular Probes).

Propidium iodide (PI) was used for labelling dead cells. Cultures washed in PBS were labelled with PI at 3 µM.

Cells either labelled with CTC-SYTO24 or PI were wet mounted onto a glass slide and viewed at ×1,000 magnification using Axioskop microscope (Zeiss). At least 1000 cells were counted per condition.

For CFU counts, cells in mid log were diluted 100 times in MHB-CA, and incubated 10 mins at 37 °C before they were plated on TSA for CFU count.

Intracellular ATP determination

To determine *P.aeruginosa* intracellular ATP content, ATP Determination Kit (Life technologies, Grand Island, NY, USA) was used. The same preparation as for CFU count was made and the cells were then washed in 100 mM Tris-HCl buffer, pH 7.8. ATP quantification was done as previously described (21). One hundred microliters of the bacterial preparation was mixed with 900 µL of boiling buffer (100 mM Tris HCl, 4 mM EDTA, pH 7.8). Theses mixtures were incubated for 2 min at 100 °C and centrifuged at 9600× g for 2 min. One hundred microliters of each sample was then assayed in triplicates with SpectraMax®M3 548 Microplate Reader in luminescence mode.

Measurement of intracellular pH

The probe 2,5-Pyrrolidinedione, 1-[[[3',6'-bis(acetyloxy)-3-oxospiro[isobenzofuran-1(3H),9'-[9H]xanthen]-5(or 6)-yl]carbonyl]oxy] cFSE (Life technologies, Grand Island, NY, USA) was used for the evaluation of the intracellular pH according to manufacturer recommendations and Breeuwer et al. 1995 (22). Briefly, cultures in mi-log were washed in buffer containing 50 mM Hepes, 5 mM EDTA, pH 8 and loaded with cFSE at 5 µM. After 10 min of incubation at 37 °C with the different compounds, the loaded cells were partictioned in a 96 well plate and the fluorescence of the probe was read at 530 nm after an

excitation at 485 nm. At the end of the experiment, the extracellular fluorescence signal was determined after filtration of the cell suspension through a 0.22 µm pore size membrane filtrate.

Growth rate

Precultures were diluted 100 times in MHB-CA culture medium and dispersed in a 24 well culture plate. diNn, colistin, gentamicin, and neamine were added at different concentrations. The plates were incubated in SpectraMax®M3 548 Microplate Reader at 37 °C for 12 hours. The optical density was recorded each 30 min.

At the exponential phase, the optical density is proportional to the biomass; therefore Y values could be replaced by the optical density (23-25).

At a time interval dt, where N is the number of cells at a time t:

$$\frac{dN}{dt} = \mu N \ (1)$$

Were µ is the growth rate (min^{-1}).

(1) could be expressed as the following:

$$\frac{dN}{N} = \mu dt \ (2)$$

With $$\int \frac{dN}{N} = \int \mu dt \ (3)$$

Therefore $$LnN = \mu t + constant \ (4)$$

At t=t0, N=N0, LnN0=constant:

$$LnN = \mu t + LnN0 \ (5)$$

At the exponential phase, the number of cells is proportional to the biomass and optical density OD; (5) at the exponential phase will give (26):

$$LnOD = \mu t + LnOD_0$$

In order to determine the growth rate of *P. aeruginosa* in each condition, Ln of the OD in each condition was plotted as a function of time (min) and the exponential phase was simulated as a linear function (17) where the slope represents the growth rate µ (min^{-1}).

II. SI FIGURES

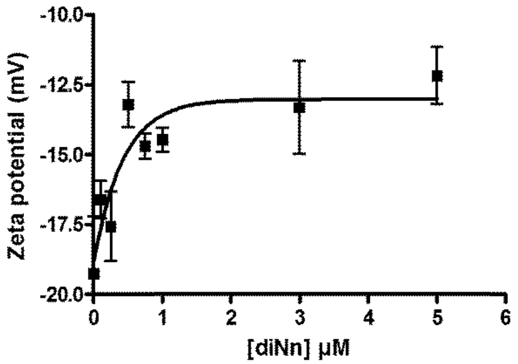

Fig. S1. Zeta potential of PE/PG/CL liposomes in the presence of increasing concentrations of diNn. (N=3, values are mean ± SEM)

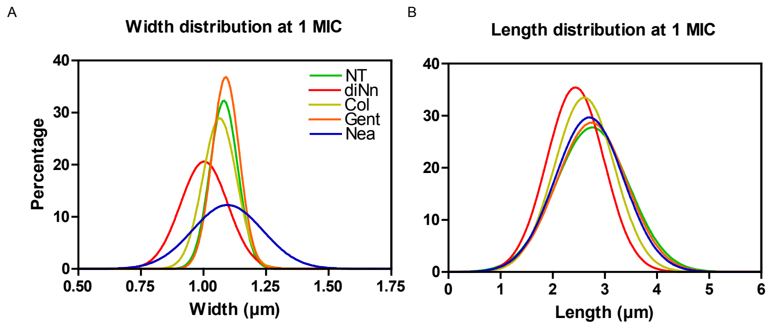

Fig. S2. Time lapse studies were conducted for 5 hours at 37 °C in CaMHB agarose pad supplemented with the different antibiotics. (A) Impact of diNn on *P. aeruginosa* width and (B) length in comparison to Colistin (Col), gentamicin (Gent), and neamine (Nea); the MICs being 1 µg/mL for colistin and gentamicin. (N≥3)

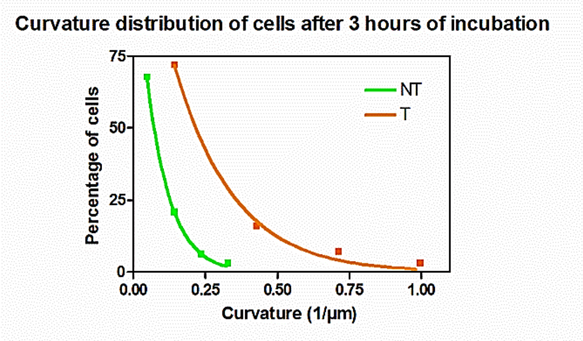

Fig. S3. Global curvature of bacterial cells after 3 hours of incubation in the absence (NT) or presence (T) of diNn at its MIC (7 μM)

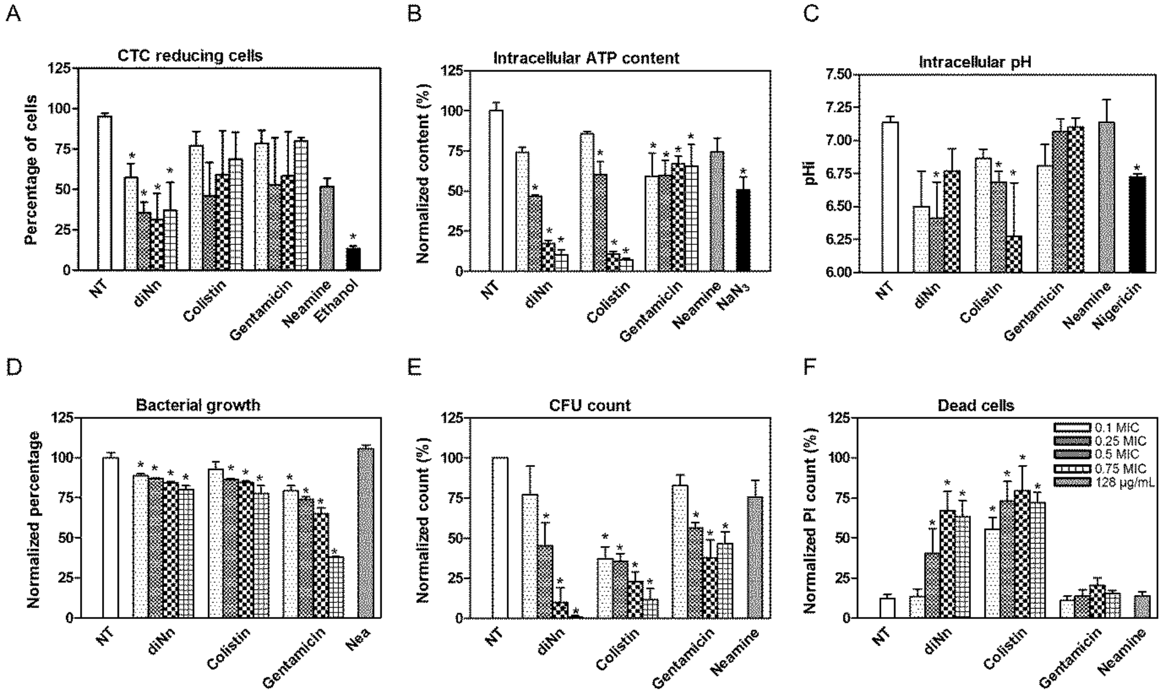

Fig. S4. Effect of diNn on the bacterial respiratory chain after 10 min of incubation in comparison to colistin, gentamicin, and neamine: (A) evaluation of the redox chain through the reduction of the 5-cyano-2,3-ditolyl tetrazolium chloride CTC, (B) analysis of the bacterial intracellular content of ATP_i, (C) measurement of the bacterial intracellular pH_i, (D) bacterial growth rate, (E) CFU, and (F) dead cells counts. All parameters are expressed in percentage when compared to the control condition. Experiments were performed at least three times in triplicate. Values are mean ≥ SEM. *, $P < 0.05$; in comparison to the untreated control cells. Ethanol, natrium azide, and nigericine were used as positive controls for redox chain inhibition, decrease of ATPi content and decrease in pHi, respectively.

I. SI MOVIES

SM. 1. Time lapse of *Pseudomonas aeruginosa* producing mCherry-MreB deposited on LB-agarose pad showing the distribution and movement of MreB in cells

SM. 2. Time lapse of *Pseudomonas aeruginosa* producing mCherry-MreB deposited on LB-agarose pad supplemented with diNn showing the diffuse passive distribution of MreB

1. Cowles KN & Gitai Z (2010) Surface association and the MreB cytoskeleton regulate pilus production, localization and function in Pseudomonas aeruginosa. *Molecular microbiology* 76(6):1411-1426.
2. Sautrey G*, et al.* (2016) Negatively Charged Lipids as a Potential Target for New Amphiphilic Aminoglycoside Antibiotics: A BIOPHYSICAL STUDY. *The Journal of biological chemistry* 291(26):13864-13874.
3. Epand RM, Rotem S, Mor A, Berno B, & Epand RF (2008) Bacterial membranes as predictors of antimicrobial potency. *Journal of the American Chemical Society* 130(43):14346-14352.
4. Murzyn K, Rog T, & Pasenkiewicz-Gierula M (2005) Phosphatidylethanolamine-phosphatidylglycerol bilayer as a model of the inner bacterial membrane. *Biophysical journal* 88(2):1091-1103.
5. Lins,L., Brasseur,R., Malaisse,W.J., Biesemans,M., Verheyden,P., and Willem,R. (1996) Importance of the hydrophobic energy: structural determination of a hypoglycemic drug of the meglitinide family by nuclear magnetic resonance and molecular modeling Biochem.Pharmacol. 52: 1155-1168.
6. Brasseur,R., Killian,J.A., De Kruijff,B., and Ruysschaert,J.M. (1987) Conformational analysis of gramicidin-gramicidin interactions at the air/water interface suggests that gramicidin aggregates into tube-like structures similar as found in the gramicidin-induced hexagonal HII phase Biochim.Biophys.Acta 903: 11-17.
7. Fa,N., Lins,L., Courtoy,P.J., Dufrene,Y., Van Der,S.P., Brasseur,R., Tyteca,D., and Mingeot-Leclercq,M.P. (2007) Decrease of elastic moduli of DOPC bilayers induced by a macrolide antibiotic, azithromycin Biochim.Biophys.Acta 1768: 1830-1838.
8. Bensikaddour,H., Snoussi,K., Lins,L., Van Bambeke,F., Tulkens,P.M., Brasseur,R., Goormaghtigh,E., and Mingeot-Leclercq,M.P. (2008) Interactions of ciprofloxacin with DPPC and DPPG: fluorescence anisotropy, ATR-FTIR and 31P NMR spectroscopies and conformational analysis Biochim.Biophys.Acta 1778: 2535-2543
9. Deleu,M., Crowet,J.M., Nasir,M.N., and Lins,L. (2014) Complementary biophysical tools to investigate lipid specificity in the interaction between bioactive molecules and the plasma membrane: A review Biochim.Biophys.Acta 1838: 3171-3190.
10. Brasseur, R. TAMMO:theoretical analysis of membrane molecular organization. Mol.Descr.Biol.Membr.Components by Computer Conform.Anal. 203-219. 1990. CRC Press, Boca Raton, R. Brasseur.
11. Lins,L., Brasseur,R. (1995) The hydrophobic effect in protein folding FASEB J. 9: 535-540.
12. Ducarme,P., Rahman,M., and Brasseur,R. (1998) IMPALA: a simple restraint field to simulate the biological membrane in molecular structure studies Proteins 30: 357-371.
13. Jasniewski J, Cailliez-Grimal C, Younsi M, Milliere JB, & Revol-Junelles AM (2008) Fluorescence anisotropy analysis of the mechanism of action of mesenterocin 52A: speculations on antimicrobial mechanism. Applied microbiology and biotechnology 81(2):339-347.
14. Bartlett GR (1959) Phosphorus assay in column chromatography. *The Journal of biological chemistry* 234(3):466-468.
15. Sautrey G*, et al.* (2014) New amphiphilic neamine derivatives active against resistant Pseudomonas aeruginosa and their interactions with lipopolysaccharides. *Antimicrobial agents and chemotherapy* 58(8):4420-4430.
16. Beaufay F*, et al.* (2015) A NAD-dependent glutamate dehydrogenase coordinates metabolism with cell division in Caulobacter crescentus. *The EMBO journal* 34(13):1786-1800.
17. Sliusarenko O, Heinritz J, Emonet T, & Jacobs-Wagner C (2011) High-throughput, subpixel precision analysis of bacterial morphogenesis and intracellular spatio-temporal dynamics. *Molecular microbiology* 80(3):612-627.

18. Paintdakhi A*, et al.* (2015) Oufti: An integrated software package for high-accuracy, high-throughput quantitative microscopy analysis. *Molecular microbiology*.
19. Ranjit DK & Young KD (2013) The Rcs stress response and accessory envelope proteins are required for de novo generation of cell shape in Escherichia coli. *Journal of bacteriology* 195(11):2452-2462.
20. Sieracki ME, Cucci TL, & Nicinski J (1999) Flow cytometric analysis of 5-cyano-2,3-ditolyl tetrazolium chloride activity of marine bacterioplankton in dilution cultures. *Applied and environmental microbiology* 65(6):2409-2417.
21. Runti G*, et al.* (2013) Functional characterization of SbmA, a bacterial inner membrane transporter required for importing the antimicrobial peptide Bac7(1-35). *Journal of bacteriology* 195(23):5343-5351.
22. Breeuwer P, Drocourt J, Rombouts FM, & Abee T (1996) A Novel Method for Continuous Determination of the Intracellular pH in Bacteria with the Internally Conjugated Fluorescent Probe 5 (and 6-)-Carboxyfluorescein Succinimidyl Ester. *Applied and environmental microbiology* 62(1):178-183.
23. Hosein AM, Breidt F, Jr., & Smith CE (2011) Modeling the effects of sodium chloride, acetic acid, and intracellular pH on survival of Escherichia coli O157:H7. *Applied and environmental microbiology* 77(3):889-895.
24. Kim DJ, Choi JW, Choi NC, Mahendran B, & Lee CE (2005) Modeling of growth kinetics for Pseudomonas spp. during benzene degradation. *Applied microbiology and biotechnology* 69(4):456-462.
25. Hall BG, Acar H, Nandipati A, & Barlow M (2014) Growth rates made easy. *Molecular biology and evolution* 31(1):232-238.
